# Supplementary material for: Effectiveness and cost-effectiveness of a cardiovascular risk prediction algorithm for people with severe mental illness (PRIMROSE)
Source: BMJ Open. 2017 Sep 5;7(9):e018181. doi: 10.1136/bmjopen-2017-018181 (PMC5588956; doi:10.1136/bmjopen-2017-018181)
Supplement: Supplementary file 1 [file bmjopen-2017-018181supp001.pdf]

Supplementary Appendices

eResults 1

eResults 1.1 Sub-analysis

eResults 1.2 Deterministic analysis

eFigures

eFigure 1: Cost-effectiveness acceptability curves of each CVD risk algorithm, compared to no algorithm, when a CVD risk threshold of 20% was employed.

eFigure 2: Cost-effectiveness planes of the head-to-head comparisons of each CVD risk algorithm sub-type when a CVD risk threshold of 10% was employed where a) compares the SMI-specific lipid algorithm versus the general lipid algorithm and b) compares the SMI-specific BMI algorithm versus the general BMI algorithm.

eFigure 3: Cost-effectiveness planes of the head-to-head comparisons of each CVD risk algorithm sub-type when a CVD risk threshold of 20% was employed where a) compares the SMI-specific lipid algorithm versus the general lipid algorithm and b) compares the SMI-specific BMI algorithm versus the general BMI algorithm.

eTables

eTable 1: Co-efficients for the a) SMI-specific and b) general algorithms.

eTable 2: Co-efficients of the covariates included in the Weibull models for primary CVD events (CHD and CVA) and death from other causes.

eTable 3: Percentage of patients who had a non-fatal or fatal primary and secondary CVD event, stratified by type of CVD event.

eTable 4: Data inputs used in our Markov model.

eTable 5: Number of people (out of 1000) classified as high and low risk by the various CVD risk algorithms at a CVD risk threshold of 20%; further stratified by use of statin therapy at baseline.

eTable 6: Costs, QALYs, NMBs and number of events prevented per 1000 individuals for each CVD algorithm (including no algorithm) when a CVD risk threshold of 20% was employed.

eTable 7: Number of events per 1000 individuals, stratified by risk and statin therapy at baseline, for each CVD algorithm (including no algorithm) when a CVD risk threshold of a) 10% and b) 20% was employed.

eTable 8: Deterministic analyses when a) the mean values for all variables are held (the base case deterministic analysis), b) all costs are doubled, c) CVD risk management with statin therapy costs are doubled, d) CVD risk algorithm costs are doubled, e) cardiovascular event costs are doubled, f) the utility for severe mental illness (SMI) is altered to the utility associated with relapse, g) the utility for SMI is altered to the utility associated with extra pyramidal symptoms (EPS), h) the effect of statin therapy is reduced to the upper odds ratio of the 95% confidence interval and i) compliance with statin therapy is reduced to 50%.

eResults 1

eResults 1.1 Sub-analysis

Head-to-head comparisons of the general and SMI-specific sub-type algorithms demonstrated the general lipid algorithm was more favourable than the SMI-specific lipid algorithm as the difference in costs between the SMI-specific algorithm and the general algorithm was greater. That is, the SMI-specific lipid algorithm had greater total costs than the general lipid algorithm. This, however, was not the case for the BMI versions, where the SMI-specific BMI algorithm performed better as the difference in costs between the SMI-specific algorithm and the general algorithm was less. The results were similar for both the 10% and 20% CVD risk thresholds, depicted in eFigure 2 and eFigure 3 respectively.

eResults 1.2 Deterministic analysis

The base case deterministic analysis (eTable 8a) represents the results when all input parameters are held at their mean value. In line with our PSA analysis, the base case deterministic analysis demonstrated greater cost-effectiveness with the SMI-specific BMI algorithm than other algorithms, with a NMB of £138,325,600 at a WTP per QALY gained of £20,000. Variation of individual input parameters produced different results for the most cost-effective algorithm. When compliance with statin therapy was reduced to 50%, the general lipid algorithm had the highest NMB. Altering the effectiveness of statin therapy by reducing its treatment effect resulted in the SMI-specific BMI algorithm performing better. Analyses where all costs were doubled resulted in the SMI-specific BMI algorithm with the highest NMB. This was also the case when only CVD risk algorithm costs were doubled. When only the costs of CVD risk management were doubled or only the costs of cardiovascular events were doubled, the SMI-specific BMI algorithm and general lipid algorithm were comparable. Reducing the utility associated with SMI to the utility associated with SMI and extra pyramidal symptoms (EPS) or to the utility associated with relapse, the general lipid algorithm was most cost-effective. In all analyses, the SMI-specific BMI algorithm or general lipid algorithm were superior to other CVD risk algorithms assessed. Differences in NMB between the SMI-specific BMI algorithm and general lipid algorithm were minimal. Results of the one-way deterministic analyses are reported in eTable 8.

eFigure 1: Cost-effectiveness acceptability curves of each CVD risk algorithm, compared to no algorithm, when a CVD risk threshold of 20% was employed.

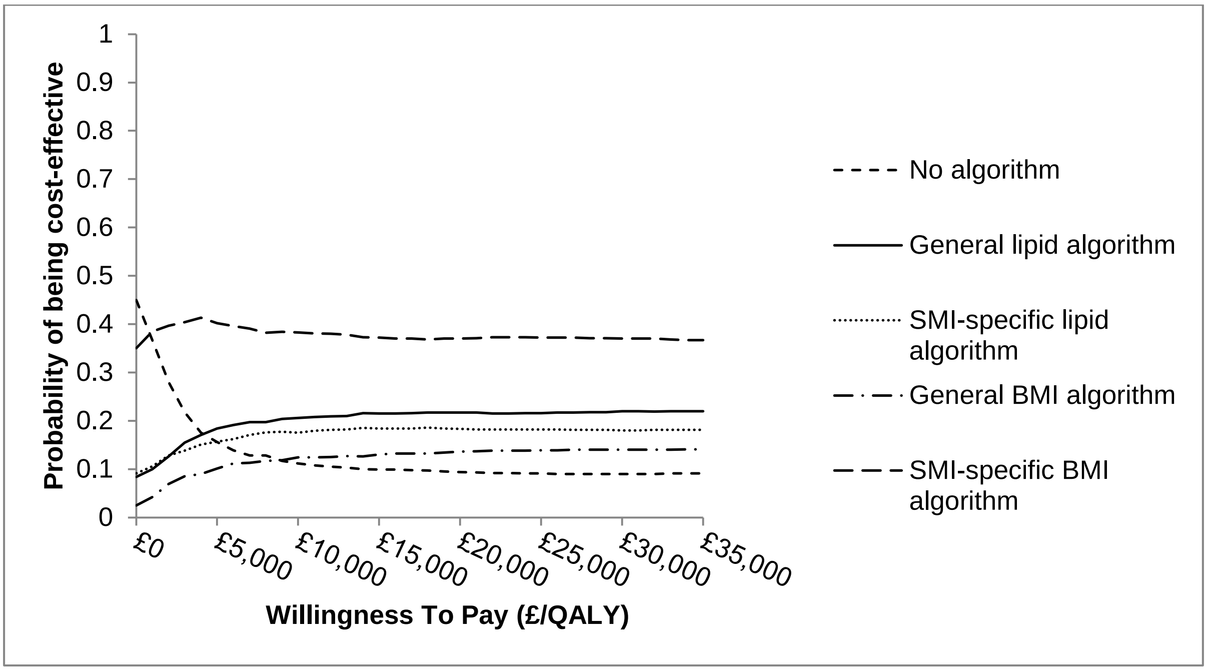

eFigure 2: Cost-effectiveness planes of the head-to-head comparisons of each CVD risk algorithm sub-type when a CVD risk threshold of 10% was employed where a) compares the SMI-specific lipid algorithm versus the general lipid algorithm and b) Compares the SMI-specific BMI algorithm versus the general BMI algorithm.

a) SMI-specific lipid algorithm versus general lipid algorithm

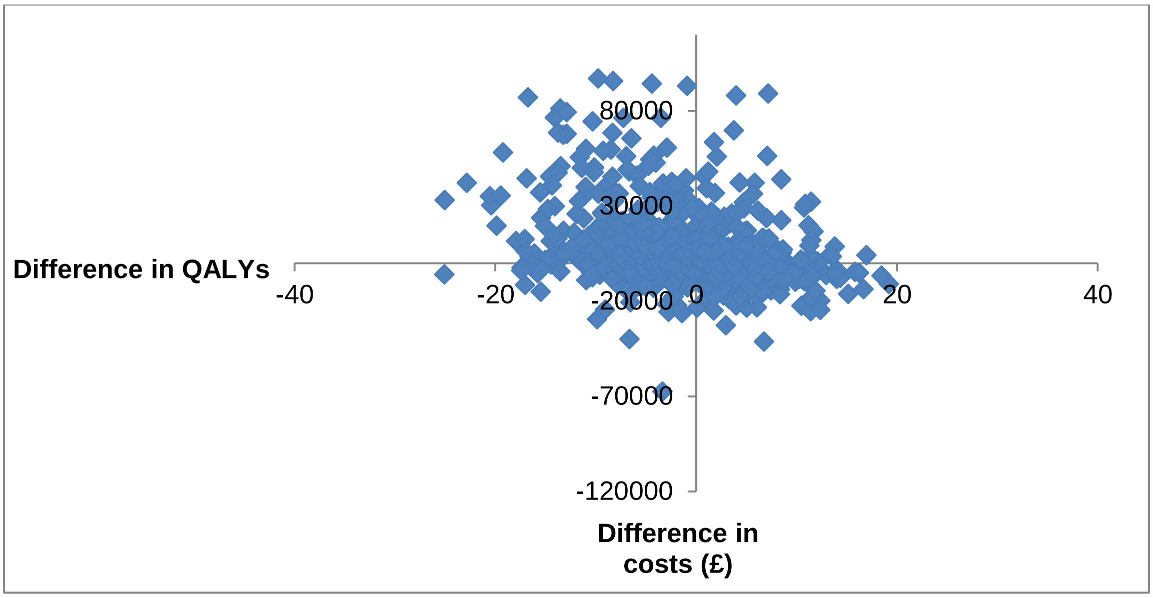

b) SMI-specific BMI algorithm versus general BMI algorithm

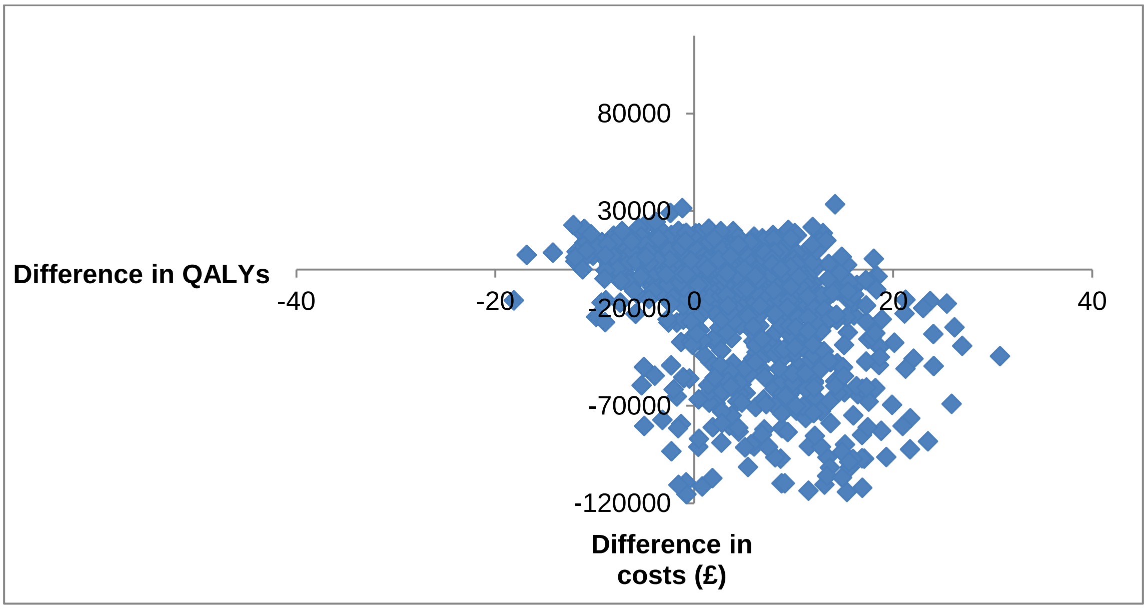

eFigure 3: Cost-effectiveness planes of the head-to-head comparisons of each CVD risk algorithm sub-type when a CVD risk threshold of 20% was employed where a) compares the SMI-specific lipid algorithm versus the general lipid algorithm and b) compares the SMI-specific BMI algorithm versus the general BMI algorithm.

a) SMI-specific lipid algorithm versus general lipid algorithm

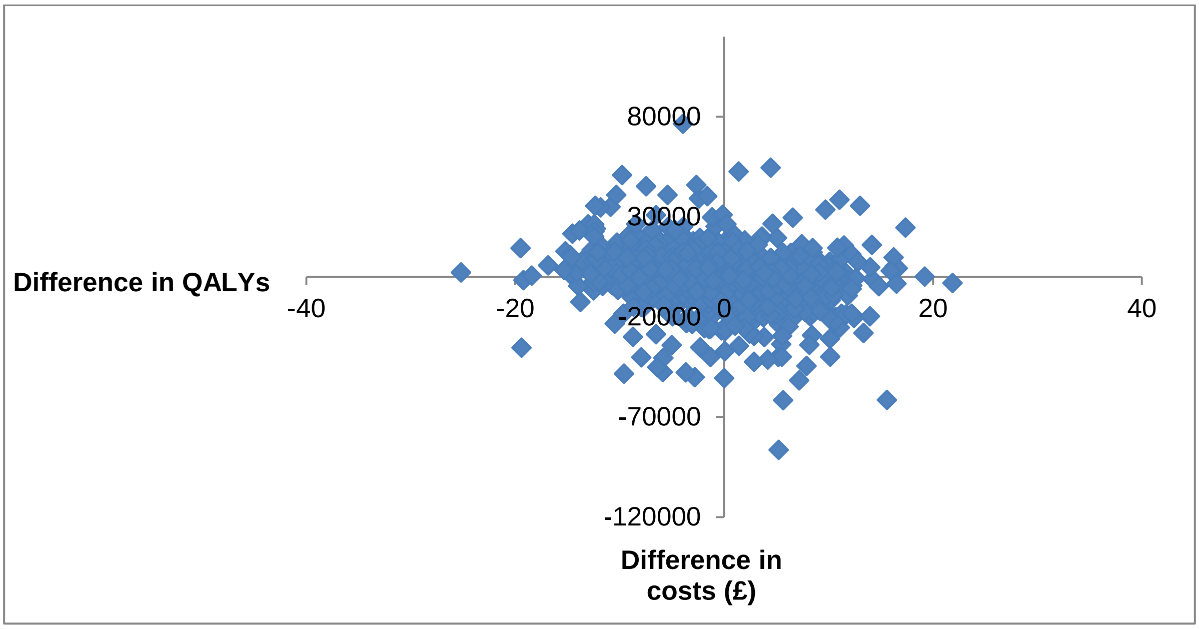

b) SMI-specific BMI algorithm versus the general BMI algorithm

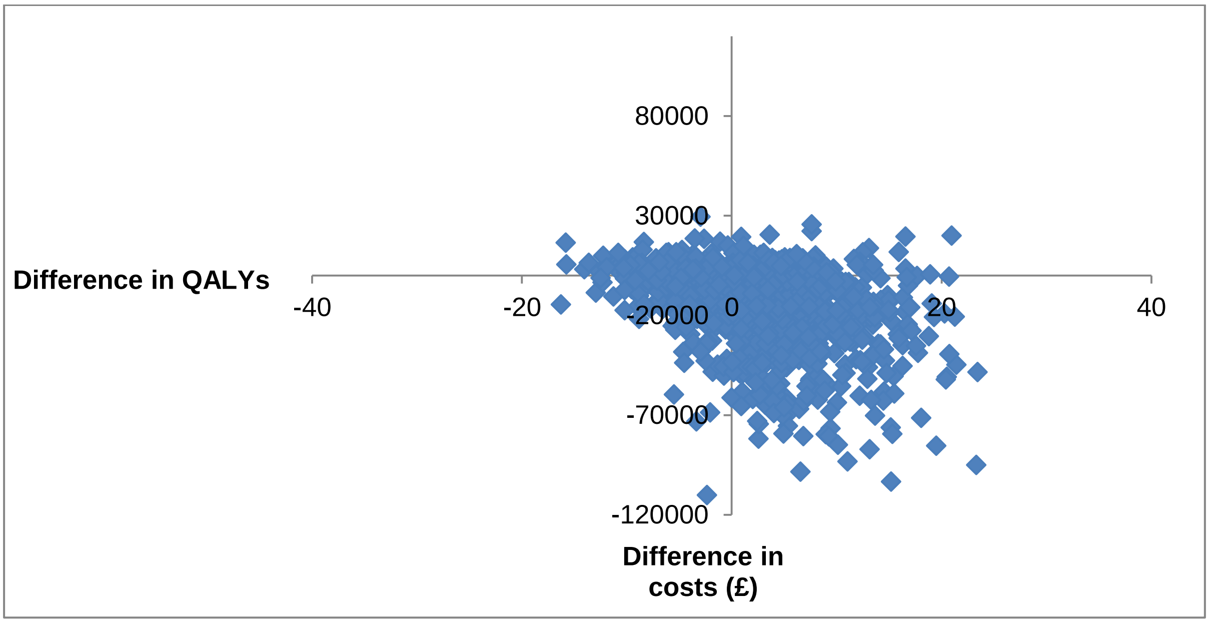

eTable 1: Co-efficients for the a) SMI-specific and b) general algorithms.

a) Co-efficients for the SMI-specific algorithms

Key to symbols in formulae:

**Symbol**	**Evaluate for patient as follows:**
fem	Takes value 1 if female; takes value 0 if male
age	Years
sbp	Systolic blood pressure (mmHg)
hyp	Takes value 1 if use of anti-hypertensives = yes; takes value 0 if use of anti-hypertensives = no
chol	Total cholesterol (mmol/L)
HDL	HDL cholesterol (mmol/L)
wt	Weight (kg)
ht	Height (cm)
dm	Takes value 1 if diabetes = yes; takes value 0 if diabetes = no
ex	Takes value 1 if ex-smoker; takes value 0 otherwise
curr	Takes value 1 if current smoker; takes value 0 otherwise
dep	Takes value 1 if use of antidepressants = yes; takes value 0 if use of antidepressants = no
alc	Takes value 1 if history of heavy drinking = yes; takes value 0 if history of heavy drinking = no
t2	Takes value 1 if patient belongs to Townsend quintile 2; takes value 0 otherwise
t3	Takes value 1 if patient belongs to Townsend quintile 3; takes value 0 otherwise
t4	Takes value 1 if patient belongs to Townsend quintile 4; takes value 0 otherwise
t5	Takes value 1 if patient belongs to Townsend quintile 5; takes value 0 otherwise
bip	Takes value 1 if patient has bipolar disorder; takes value 0 otherwise
oth	Takes value 1 if patient has psychosis other than schizophrenia or bipolar disorder; takes value 0 otherwise
reg	Takes value 1 if has unspecified SMI but has been added to an SMI register; takes value 0 otherwise
atyp	Takes value 1 if use of atypical (second generation) antipsychotics = yes; takes value 0 if use of atypical antipsychotics = no
typ	Takes value 1 if use of typical (first generation) antipsychotics = yes; takes value 0 if use of typical antipsychotics = no
cal	Calendar year e.g. 2013

Covariates	Lipid model	BMI model
fem	- 0.1795	- 0.49376
age	3.78124×(log(age) – 3.853361)	3.50943×(log(age) – 3.853361)
sbp	0.007651× (SBP – 129.8673)	0.00893×(sbp – 129.8673)
hyp	0.625719	0.65817264
ln(hyp) x lnsbp	- 0.00796×hyp×(sbp – 129.8673)	- 0.00888×hyp×(SBP – 129.8673)
chol	0.11763×(chol – 5.562413)	
HDL	- 0.8183×(HDL – 1.389071)	
wt		0.000680×(wt – 76.20105)
ht		- 0.0124×(ht – 167.9494)
dm	0.37734	0.44971
ex	0.01639	0.0738
curr	0.29659	0.38081
dep	0.2104	0.21846
alc	0.41392	0.30721
t2	0.10963	0.10919
t3	0.16388	0.18412
t4	0.1828	0.20238
t5	0.22126	0.24762
bip	0.11177	0.0978
oth	0.21004	0.19063
reg	0.01526	- 0.01138
atyp	0.12121	0.17662
typ		0.1205
cal	- 0.07043×(cal -2001.83)	- 0.07524×(cal -2001.83)
S	0.968011	0.951285

Then predicted risk (as a percentage) = 100×[1-$S^{exp(P)}$] where P=sum(fem + age + sbp...)

b) Co-efficients for the general algorithms.

Key to symbols in formulae:

Symbol	Evaluate for patient as follows:
lnage	ln age (years)
lnsbp	ln Systolic blood pressure (mmHg)
ln(hyp)	Takes value 1 if use of antihypertensives = yes; takes value 0 if use of antihypertensives = no
lnchol	ln Total cholesterol (mmol/L)
lnhdl	ln HDL cholesterol (mmol/L)
lnbmi	ln BMI (kg/m^2)
dm	Takes value 1 if diabetes = yes; takes value 0 if diabetes = no
curr	Takes value 1 if current smoker; takes value 0 otherwise

Where ln = natural log,

Covariates	Lipid model		BMI model	
	**Male**	**Female**	**Male**	**Female**
lnage	4.14474 x (lnage - 3.7901)	4.41759 x (lnage – 3.8289)	4.07664 x (lnage – 3.7901)	4.28501 x (lnage – 3.8289)
lnsbp	0.9025974 x (lnsbp-4.8792)	1.37844 x (lnsbp – 4.8474)	1.00391 x (lnsbp – 4.8792)	1.69393 x (lnsbp – 4.8474)
ln(hyp)	0.458338 x ln(hyp)	0.552473 x ln(hyp)	0.489864 x ln(hyp)	0.627038 x ln(hyp)
ln(hyp) x lnsbp	-0.840329 x ln(hyp) x (lnsbp-4.8792)	-0.786195 x ln(hyp) x (lnsbp – 4.8474)	-0.882539 x ln(hyp) x (lnsbp – 4.8792)	-0.929636 x ln(hyp) x (lnsbp – 4.8474)
lnchol	0.78074 x (lnchol – 5.3156)	0.675028 x ln(chol – 5.3388)		
lnhdl	-1.2378 x (lnhdl – 3.8676)	-1.05329 x (lnhdl – 4.0760)		
lnbmi			0.324059 x (lnbmi – 3.2744)	-0.198854 x (lnbmi – 3.2503)
dm	0.391873	0.66555	0.428547	0.796977
curr	0.204347	0.34367	0.291835	0.403768
S	0.966189	0.9833206	0.9650041	0.9824454

Then predicted risk (as a percentage) = 100×[1-$S^{exp(P)}$] where P=sum(lnage + lnsbp + hyp…)

eTable 2: Coefficient's of the covariates included in the Weibull models for primary CVD events (CHD and CVA) and death from other causes.

Covariates	Outcome					
	CHD		CVA		Death from other causes	
	Co-efficient	Standard Error	Co-efficient	Standard Error	Co-efficient	Standard Error
Age, years	0.050**	0.003	0.072**	0.003	0.075**	0.001
Female	-0.764**	0.103	-0.004	0.130	-0.399**	0.035
SBP, mmHg	0.006*	0.002	0.011**	0.002		
Anti-hypertensive therapy	0.629**	0.077	0.296**	0.074		
Total cholesterol, mmol/L	0.282**	0.061	0.043	0.062		
HDL-cholesterol, mmol/L	-0.771*	0.268	-1.108*	0.417		
Lipid lowering therapy	1.287**	0.185	1.241**	0.202		
Weight, kg	-0.002	0.003	-0.015*	0.005		
Height, m	-1.590**	0.514	-0.399	0.681		
Presence of diabetes	0.492**	0.118	0.171	0.132		
Smoking status						
Ex-smoker	0.113	0.090	-0.021	0.089		
Current smoker	0.416**	0.080	0.176*	0.087		
Depression or on anti-depressant therapy	0.194**	0.069	0.123	0.064		
History of heavy drinking	0.327**	0.117	0.380**	0.134		
Type of SMI						
Schizophrenia	0.006	0.107	-0.051	0.114		
Bipolar disorder	-0.017	0.109	0.241*	0.109		
Other non-organic psychosis	0.212*	0.104	0.165	0.109		
First generation anti-psychotic therapy	0.045	0.074	0.270**	0.071		
Second generation anti-psychotic therapy	-0.303**	0.102	0.129	0.093		
Constant	-6.984**	0.963	-8.614**	1.196	-8.719**	0.090
Gamma	1.058	0.027	1.151	0.029	1.213	0.017

Where * represents $p < 0.05$ and ** represents $p < 0.01$.

eTable 3: Proportion (%) of patients who had a non-fatal or fatal primary and secondary CVD event, stratified by type of CVD event.

**CVD event**		**No. (%)**	**Sub-type of each CVD event**	**No. (%)**
**Primary**				
CHD	Non-fatal	1002/1130 (88.7)	*Unstable angina*	65/1130 (5.8)
			Stable angina	325/1130 (28.8)
			MI	414/1130 (36.6)
			Surgery	22/1130 (1.9)
			Unclassified CHD	304/1130 (26.9)
	Fatal	128/1130 (11.3)		
CVA	Non-fatal	1111/1194 (93)	*TIA*	349/1194 (29.2)
			Haemorrhagic stroke	46/1194 (3.9)
			Ischaemic stroke	778/1194 (65.2)
			Unspecified stroke	21/1194 (1.8)
	Fatal	83/1194 (7)		
**Secondary**				
CVD	Non-fatal	3166/5481 (57.8)	*CHD*	1228/3166 (38.8)
			CVA	1898/3166 (59.9)
			CHD and CVA	40/3166 (1.3)
	Fatal	2315/5481 (42.2)	*CHD*	1157/2315 (50)
			CVA	1158/2315 (50)

eTable 4: Data inputs used in our Markov model.

Input variables	Utilities			Costs (£) First year			Costs (£) Subsequent year			Effect of statin therapy		
	Mean	Standard Error	Distribution	Mean	Standard Error	Distribution	Mean	Standard Error	Distribution	Mean	Standard Error	Distribution
SMI	0.865	0.021	beta									
CVD event												
CHD										0.73	0.033	log
Unstable angina	-0.216	0.014	gamma	£566	£566	gamma	£220	£220	gamma			
Stable angina	-0.216	0.014	gamma	£220	£220	gamma	£220	£220	gamma			
MI	-0.072	0.005	gamma	£5,720	£5,720	gamma	£220	£220	gamma			
Surgery	-0.072	0.005	gamma	£6,008	£6,008	gamma	n/a					
Unclassified CHD	-0.101	0.006	gamma	£2,169	£2,169	gamma	£220	£220	gamma			
Fatal CHD	n/a			£1,500	£1,500	gamma	n/a					
Stroke										0.78	0.054	log
TIA	-0.088	0.006	gamma	£1,368	£1,368	gamma	£340	£340	gamma			
Stroke	-0.185	0.012	gamma	£10,347	£10,347	gamma	£2,782	£2,782	gamma			
Unspecified CVA	-0.153	0.01	gamma	£5,858	£5,858	gamma	£1,561	£1,561	gamma			
Fatal stroke	n/a			£9,055	£9,055	gamma	n/a					

eTable 5: Number of people (out of 1000) classified as high and low risk by the various CVD risk algorithms at a CVD risk threshold of 20%; further stratified by use of statin therapy at baseline.

	**Algorithm**			
	**General lipid algorithm**	**SMI-specific lipid algorithm**	**General BMI algorithm**	**SMI-specific BMI algorithm**
High risk (≥20%)				
Total	82	78	65	117
Currently prescribed statins	26	23	21	36
Not currently prescribed statins	56	55	44	81
Low risk (<20%)				
Total	918	922	935	883
Currently prescribed statins	71	74	76	61
Not currently prescribed statins	847	848	859	822

eTable 6: Costs, QALYs, NMBs and number of events prevented per 1000 individuals for each CVD algorithm (including no algorithm) when a CVD risk threshold of 20% was employed.

	Algorithm				
**Outcomes**	**General lipid algorithm**	**SMI-specific lipid algorithm**	**General BMI algorithm**	**SMI-specific BMI algorithm**	**No algorithm**
Costs and QALYs, mean (95% CI)					
Costs of administering algorithm	£19,925 (19,831 - 20,019)	£19,925 (19,831 - 20,019)	£18,960 (18,886 - 19,033)	£18,960 (18,886 - 19,033)	n/a
Costs of new statin prescriptions	£10,362 (10,227 - 10,497)	£10,357 (10,222 - 10,491)	£8,045 (7,940 - 8,151)	£14,907 (14,712 - 15,102)	n/a
Costs of CVD events	£1,810,519 (1,653,359 - 1,967,680)	£1,809,957 (1,652,869 - 1,967,046)	£1,819,735 (1,662,575 - 1,976,894)	£1,797,005 (1,640,538 - 1,953,472)	£1,845,719 (1,687,645 - 2,003,793)
Total costs undiscounted	£1,840,807 (1,683,666 - 1,997,947)	£1,840,239 (1,683,171 - 1,997,306)	£1,846,740 (1,689,595 - 2,003,885)	£1,830,872 (1,674,429 - 1,987,315)	£1,845,719 (1,687,645 - 2,003,793)
Total costs discounted	£1,587,688 (1,451,812 - 1,723,564)	£1,587,306 (1,451,485 - 1,723,127)	£1,592,802 (1,456,909 -1,728,696)	£1,578,939 (1,443,689 - 1,714,188)	£1,589,781 (1,453,019 - 1,726,543)
QALY discounted	6,823 (6,809 - 6,837)	6,823 (6,809 - 6,837)	6,822 (6,808 - 6,836)	6,825 (6,811 - 6,838)	6,819 (6,805 - 6,833)
Cost compared to no algorithm	-£2,093	-£2,476	£3,021	-£10,483	
QALY compared to no algorithm	4	3	4	6	
Net monetary benefit, mean (95% CI)					
£20,000 WTP threshold	134,876,090 (134,486,620 - 135,265,560)	134,868,737 (134,479,388 - 135,258,085)	134,843,556 (134,453,878 - 135,233,235)	134,913,127 (134,526,041 - 135,300,214)	134,785,088 (134,392,268 - 135,177,908)
£30,000 WTP threshold	203,107,979 (202,583,230 - 203,632,729)	203,096,758 (202,572,212 - 203,621,303)	203,061,735 (202,536,664 - 203,586,807)	203,159,160 (202,637,681 - 203,680,639)	202,972,522 (202,443,200 - 203,501,845)

eTable 6: Costs, QALYs, NMBs and number of events prevented per 1000 individuals for each CVD algorithm (including no algorithm) when a CVD risk threshold of 20% was employed (continued).

	Algorithm				
**Outcomes**	**General lipid algorithm**	**SMI-specific lipid algorithm**	**General BMI algorithm**	**SMI-specific BMI algorithm**	**No algorithm**
Events, mean (95% CI)					
Primary non-fatal CHD	85.36 (78.78 - 91.95)	85.47 (78.88- 92.07)	85.93 (79.32 - 92.53)	84.75 (78.20 - 91.31)	87.29 (80.63 - 93.96)
Primary fatal CHD	9.69 (8.93 - 10.44)	9.70 (8.95- 10.46)	9.73 (8.97 - 10.48)	9.61 (8.86 - 10.36)	9.90 (9.13 - 10.66)
Primary non-fatal stroke	100.68 (91.61 - 109.76)	100.69 (91.62 - 109.76)	100.97 (91.90 - 110.05)	100.11 (91.06 - 109.15)	102.00 (92.89 - 111.10)
Primary fatal stroke	7.04 (6.39 - 7.68)	7.04 (6.40 - 7.69)	7.07 (6.42 - 7.71)	7.00 (6.36 - 7.64)	7.14 (6.49 - 7.79)
Secondary non-fatal CVD	15.46 (14.41 - 16.51)	15.46 (14.41 - 16.51)	15.70 (14.65 - 16.76)	15.21 (14.18 - 16.25)	16.34 (15.26 - 17.43)
Secondary fatal CVD	6.91 (6.47 - 7.35)	6.92 (6.48 - 7.37)	7.01 (6.56 - 7.46)	6.78 (6.35 - 7.22)	7.41 (6.94 - 7.87)
Death from other causes	119.58 (118.75 - 120.40)	119.67 (118.84 - 120.50)	119.61 (118.78 - 120.44)	119.63 (118.80 - 120.46)	119.42 (118.60 - 120.25)

QALYs are the quality adjusted life years, NMB is the net monetary benefit, WTP is the willingness to pay, and discounted costs and QALYs reflect time-preference for current benefits over future ones.

eTable 7: Number of events per 1000 individuals, stratified by risk and statin therapy at baseline, for each CVD algorithm when a CVD risk threshold of a) 10% and b) 20% was employed.

a) 10% CVD risk threshold

Outcomes	Algorithm			
	**General lipid algorithm**	**SMI-specific lipid algorithm**	**General BMI algorithm**	**SMI-specific BMI algorithm**
**High risk, mean (95% CI)**				
Statin therapy				
Primary non-fatal CHD	311.38 (295.77 - 326.98)	311.40 (295.79 - 327.01)	301.08 (285.92 - 316.24)	288.18 (273.13 - 303.04)
Primary fatal CHD	35.23 (33.44 - 37.02)	35.26 (33.49 - 37.02)	34.07 (32.34 - 35.79)	32.67 (30.97 - 34.37)
Primary non-fatal stroke	324.93 (305.97 - 343.88)	324.55 (305.55 - 343.55)	325.12 (305.96 - 344.27)	307.04 (288.51 - 325.57)
Primary fatal stroke	22.59 (21.25 - 23.93)	22.44 (21.11 - 23.78)	22.56 (21.20 - 23.91)	21.28 (19.98 - 22.58)
Secondary non-fatal CVD	302.33 (284.70 - 319.97)	302.11 (284.42 - 319.79)	302.56 (284.74 - 320.38)	285.76 (268.51 - 303.01)
Secondary fatal CVD	35.79 (34.42 - 37.17)	34.95 (33.63 - 36.27)	38.91 (37.42 - 40.41)	31.59 (30.33 - 32.84)
Dead from other causes	266.73 (225.11 - 228.35)	219.70 (218.12 - 22.28)	248.78 (247.03 - 250.53)	208.46 (206.98 - 209.95)
No statin therapy				
Primary non-fatal CHD	109.72 (101.22 - 118.21)	113.65 (104.99 - 122.32)	106.01 (97.77 - 114.25)	98.25 (90.47 - 106.03)
Primary fatal CHD	12.39 (11.43 - 13.35)	12.81 (11.84 - 13.79)	11.98 (11.05 - 12.91)	11.08 (10.21 - 11.96)
Primary non-fatal stroke	153.65 (139.92 - 167.37)	160.23 (146.22 - 174.25)	153.09 (139.39 - 166.79)	142.12 (129.11 - 155.14)
Primary fatal stroke	10.68 (9.71 - 11.64)	11.15 (10.17 - 12.14)	10.67 (9.71 - 11.63)	9.88 (8.96 - 10.79)
Secondary non-fatal CVD	19.98 (18.49 - 21.47)	21.12 (19.57 - 22.66)	20.08 (18.57 - 21.59)	18.05 (16.66 - 19.43)
Secondary fatal CVD	10.07 (9.32 - 10.82)	10.63 (9.85 - 11.40)	10.73 (9.92 - 11.53)	8.93 (8.25 - 9.62)
Dead from other causes	248.57 (246.91 - 250.22)	242.83 (241.21 - 244.46)	274.54 (272.73 - 276.35)	225.35 (223.83 - 226.87)

eTable 7: Number of events per 1000 individuals, stratified by risk and statin therapy at baseline, for each CVD algorithm when a CVD risk threshold of a) 10% and b) 20% was employed (continued).

a) 10% CVD risk threshold (continued)

	Algorithm			
**Outcomes**	**General lipid algorithm**	**SMI-specific lipid algorithm**	**General BMI algorithm**	**SMI-specific BMI algorithm**
**Low risk, mean (95% CI)**				
Statin therapy				
Primary non-fatal CHD	161.07 (150.09 - 172.05)	157.89 (147.02 - 168.76)	203.76 (191.54 - 215.99)	150.17 (139.64 - 160.71)
Primary fatal CHD	18.32 (17.06 - 19.59)	17.88 (16.64 - 19.12)	23.02 (21.63 - 24.41)	16.99 (15.79 - 18.18)
Primary non-fatal stroke	190.44 (175.50 - 205.38)	186.84 (172.09 - 201.58)	219.50 (204.01 - 235.00)	171.19 (157.31 - 185.08)
Primary fatal stroke	13.27 (12.22 - 14.32)	12.93 (11.90 - 13.97)	15.26 (14.16 - 16.36)	11.74 (10.78 - 12.71)
Secondary non-fatal CVD	26.26 (24.70 - 27.81)	26.19 (24.61 - 27.76)	32.50 (30.87 - 34.14)	22.46 (21.08 - 23.83)
Secondary fatal CVD	9.93 (9.34 - 10.53)	10.47 (9.84 - 11.10)	12.54 (11.91 - 13.17)	8.56 (8.04 - 9.08)
Dead from other causes	100.62 (99.87 - 101.37)	108.19 (107.38 - 108.99)	107.34 (106.54 - 108.14)	86.32 (85.65 - 86.98)
No statin therapy				
Primary non-fatal CHD	49.77 (45.12 - 54.42)	51.98 (47.18 - 56.78)	55.07 (50.12 - 60.02)	49.13 (44.56 - 53.70)
Primary fatal CHD	5.62 (5.10 - 6.15)	5.88 (5.34 - 6.43)	6.23 (5.67 - 6.79)	5.55 (5.03 - 6.06)
Primary non-fatal stroke	65.86 (58.06 - 73.67)	68.28 (60.29 - 76.27)	71.26 (63.16 - 79.35)	63.17 (55.58 - 70.76)
Primary fatal stroke	4.58 (4.03 - 5.13)	4.74 (4.18 - 5.30)	4.96 (4.39 - 5.53)	4.39 (3.85 - 4.92)
Secondary non-fatal CVD	8.46 (7.65 - 9.28)	8.91 (8.06 - 9.75)	9.52 (8.66 - 10.39)	8.00 (7.22 - 8.77)
Secondary fatal CVD	2.82 (2.55 - 3.09)	3.11 (2.82 - 3.40)	3.19 (2.90 - 3.47)	2.57 (2.32 - 2.82)
Dead from other causes	72.38 (71.88 - 72.88)	80.63 (80.08 - 81.18)	74.52 (74.00 - 75.03)	69.27 (68.79 - 69.74)

eTable 7: Number of events per 1000 individuals, stratified by risk and statin therapy at baseline, for each CVD algorithm when a CVD risk threshold of a) 10% and b) 20% was employed (continued).

b) 20% CVD risk threshold

	**Algorithm**			
**Outcomes**	**General lipid algorithm**	**SMI-specific lipid algorithm**	**General BMI algorithm**	**SMI-specific BMI algorithm**
**High risk, mean (95% CI)**				
Statin therapy				
Primary non-fatal CHD	360.36 (343.15 - 377.58)	374.85 (357.39 - 392.30)	361.84 (344.79 - 378.88)	323.62 (307.45 - 339.79)
Primary fatal CHD	40.88 (38.90 - 42.85)	42.65 (40.64 - 44.66)	40.99 (39.03 - 42.95)	36.63 (34.77 - 38.49)
Primary non-fatal stroke	349.83 (330.33 - 369.32)	347.56 (328.05 - 367.06)	353.54 (333.87 - 373.21)	333.20 (314.14 - 352.26)
Primary fatal stroke	24.38 (22.97 - 25.79)	24.28 (22.88 - 25.68)	24.74 (23.33 - 26.16)	23.27 (21.91 - 24.63)
Secondary non-fatal CVD	325.44 (307.33 - 343.56)	323.27 (305.14 - 341.41)	328.80 (310.51 - 347.08)	309.93 (292.21 - 327.66)
Secondary fatal CVD	48.31 (46.62 - 49.99)	46.56 (45.00 - 48.12)	54.80 (52.8 7- 56.72)	42.26 (40.69 - 43.83)
Dead from other causes	279.84 (277.79 - 281.88)	261.78 (259.84 - 263.73)	305.16 (302.94 - 307.37)	254.98 (253.12 - 256.84)
No statin therapy				
Primary non-fatal CHD	153.12 (142.21 - 164.03)	153.74 (142.86 - 164.63)	137.24 (127.29 - 147.19)	136.97 (126.95 - 146.99)
Primary fatal CHD	17.32 (16.07 - 18.57)	17.41 (16.17 - 18.66)	15.56 (14.42 - 16.70)	15.47 (14.33 - 16.60)
Primary non-fatal stroke	187.67 (171.54 - 201.80)	191.00 (175.78 - 206.22)	174.64 (159.83 - 189.44)	171.87 (157.52 - 186.21)
Primary fatal stroke	13.00 (11.93 - 14.07)	13.47 (12.37 - 14.56)	12.20 (11.15 - 13.25)	12.01 (10.99 - 13.02)
Secondary non-fatal CVD	28.12 (26.32 - 29.92)	28.54 (26.73 - 30.34)	25.68 (23.96 - 27.40)	24.94 (23.27 - 26.60)
Secondary fatal CVD	15.96 (14.94 - 16.98)	15.66 (14.67 - 16.65)	16.10 (15.01 - 17.19)	13.58 (12.67 - 14.49)
Dead from other causes	323.68 (321.51 - 325.84)	298.04 (296.03 - 300.05)	383.61 (381.12 - 386.09)	292.45 (290.47 - 294.43)

eTable 7: Number of events per 1000 individuals, stratified by risk and statin therapy at baseline, for each CVD algorithm when a CVD risk threshold of a) 10% and b) 20% was employed (continued).

b) 20% risk threshold (continued)

	Algorithm			
**Outcomes**	**General lipid algorithm**	**SMI-specific lipid algorithm**	**General BMI algorithm**	**SMI-specific BMI algorithm**
**Low risk, mean (95% CI)**				
Statin therapy				
Primary non-fatal CHD	209.51 (196.60 - 222.42)	211.26 (198.26 - 224.26)	218.96 (205.76 - 232.16)	206.44 (193.67 - 219.21)
Primary fatal CHD	23.77 (22.31 - 25.23)	23.95 (22.47 - 25.42)	24.75 (23.25 - 26.24)	23.44 (21.98 - 24.90)
Primary non-fatal stroke	230.25 (214.51 - 246.00)	235.68 (219.82 - 251.54)	236.96 (221.06 - 252.86)	220.08 (204.71 - 235.46)
Primary fatal stroke	16.01 (14.89 - 17.13)	16.44 (15.31 - 17.57)	16.64 (15.50 - 17.78)	15.35 (14.26 - 16.44)
Secondary non-fatal CVD	35.99 (34.19 - 37.80)	37.90 (36.03 - 39.78)	37.72 (35.89 - 39.55)	34.06 (32.33 - 35.78)
Secondary fatal CVD	16.19 (15.38 - 17.00)	17.95 (17.07 - 18.83)	16.50 (15.70 -17.30)	14.51 (13.77 - 15.24)
Dead from other causes	138.63 (137.59 - 139.67)	149.94 (148.83 - 151.05)	141.01 (139.96 -142.06)	130.07 (129.12 - 131.03)
No statin therapy				
Primary non-fatal CHD	62.04 (56.40 - 67.67)	62.22 (56.57 - 67.87)	64.78 (59.02 -70.54)	60.12 (54.59 - 65.65)
Primary fatal CHD	7.04 (6.39 - 7.69)	7.07 (6.42 - 7.71)	7.33 (6.68 - 7.99)	6.83 (6.19 - 7.46)
Primary non-fatal stroke	76.49 (68.47 - 84.51)	76.36 (68.34 - 84.37)	78.99 (70.88 - 87.10)	73.92 (66.08 - 81.77)
Primary fatal stroke	5.36 (4.79 - 5.93)	5.34 (4.77 - 5.91)	5.52 (4.95 - 6.10)	5.18 (4.62 - 5.73)
Secondary non-fatal CVD	10.98 (10.06 - 11.89)	10.97 (10.05 - 11.88)	11.62 (10.68 - 12.55)	10.45 (9.57 - 11.34)
Secondary fatal CVD	4.26 (3.91 - 4.61)	4.32 (3.97 - 4.68)	4.54 (4.18 - 4.90)	3.99 (3.65 - 4.32)
Dead from other causes	99.56 (98.87 - 100.26)	101.60 (100.90 - 102.31)	99.66 (98.95 - 100.36)	95.90 (95.23 - 96.57)

eTable 8: Deterministic analyses when a) the mean values for all variables are held (the base case deterministic analysis), b) all costs are doubled, c) CVD risk management with statin therapy costs are doubled, d) CVD risk algorithm costs are doubled, e) cardiovascular event costs are doubled, f) the utility for severe mental illness (SMI) is altered to the utility associated with relapse, g) the utility for SMI is altered to the utility associated with extra pyramidal symptoms (EPS), h) the effect of statin therapy is reduced to the upper odds ratio of the 95% confidence interval and i) compliance with statin therapy is reduced to 50%.

a) Deterministic analyses with 5,000 iterations when the mean values for all variables are held (the base case deterministic analysis).

**Outcomes**	**Algorithm**				
	**General lipid algorithm**	**SMI-specific lipid algorithm**	**General BMI algorithm**	**SMI-specific BMI algorithm**	**No algorithm**
Costs, QALYs and number of events, mean					
Total costs discounted	£557,265	£555,615	£557,715	£558,293	£546,095
QALY discounted	6,944	6,942	6,944	6,944	6,939
Cost compared to no algorithm	£11,170	£9,520	£11,620	£12,198	
QALY compared to no algorithm	5	3	5	5	
Primary CVD	98.53	99.37	100.14	97.59	106.82
Secondary CVD	8.32	8.39	8.57	8.24	9.81
Death from other causes	119.98	120.00	119.69	119.89	120.09
Net monetary benefit per patient, mean					
£20,000 WTP threshold	£138,321,726	£138,286,240	£138,323,280	£138,325,660	£138,225,080
£30,000 WTP threshold	£207,761,2215	£207,707,167	£207,763,778	£207,767,637	£207,610,6676

QALYs are the quality adjusted life years, WTP is the willingness to pay, and discounted costs and QALYs reflect time-preference for current benefits over future ones.

b) Deterministic analyses with 5,000 iterations when all costs are doubled.

Outcomes	Algorithm				
	**General lipid algorithm**	**SMI-specific lipid algorithm**	**General BMI algorithm**	**SMI-specific BMI algorithm**	**No algorithm**
Costs, QALYs and number of events, mean					
Total costs discounted	£1,113,829	£1,110,118	£1,113,288	£1,120,755	£1,091,295
QALY discounted	6,944	6,944	6,944	6,945	6,939
Cost compared to no algorithm	£22,534	£18,823	£21,993	£29,460	
QALY compared to no algorithm	5	5	5	6	
Primary CVD	98.59	99.12	99.93	97.79	106.74
Secondary CVD	8.36	8.41	8.56	8.25	9.85
Death from other causes	119.90	119.84	119.77	119.90	119.83
Net monetary benefit per patient, mean					
£20,000 WTP threshold	£137,766,055	£137,767,426	£137,765,085	£137,772,135	£137,696,857
£30,000 WTP threshold	£207,205,997	£207,206,198	£207,204,272	207,218,580	£207,090,933

QALYs are the quality adjusted life years, WTP is the willingness to pay, and discounted costs and QALYs reflect time-preference for current benefits over future ones.

c) Deterministic analyses with 5,000 iterations when CVD risk management with statin therapy costs are doubled.

	Algorithm				
**Outcomes**	**General lipid algorithm**	**SMI-specific lipid algorithm**	**General BMI algorithm**	**SMI-specific BMI algorithm**	**No algorithm**
Costs, QALYs and number of events, mean					
Total costs discounted	£590,490	£585,669	£584,787	£600,045	£546,421
QALY discounted	6,944	6,943	6,943	6,944	6,938
Cost compared to no algorithm	£44,069	£39,248	£38,366	£53,624	
QALY compared to no algorithm	6	5	5	6	
Primary CVD	98.52	99.39	100.11	97.75	106.80
Secondary CVD	8.32	8.43	8.54	8.21	9.93
Death from other causes	119.88	120.13	119.85	119.95	119.97
Net monetary benefit per patient, mean					
£20,000 WTP threshold	£138,288,504	£138,272,245	£138,281,371	£138,284,118	£138,213,135
£30,000 WTP threshold	£207,728,000	£207,701,202	£207,714,451	£207,726,200	£207,592,913

QALYs are the quality adjusted life years, WTP is the willingness to pay, and discounted costs and QALYs reflect time-preference for current benefits over future ones.

d) Deterministic analyses with 5,000 iterations when CVD risk algorithm costs are doubled.

Outcomes	Algorithm				
	**General lipid algorithm**	**SMI-specific lipid algorithm**	**General BMI algorithm**	**SMI-specific BMI algorithm**	**No algorithm**
Costs, QALYs and number of events, mean					
Total costs discounted	£575,793	£574,462	£576,780	£579,527	£544,116
QALY discounted	6,943	6,943	6,943	6,946	6,940
Cost compared to no algorithm	£31,677	£30,346	£32,664	£35,411	
QALY compared to no algorithm	3	3	3	6	5
Primary CVD	98.71	99.16	100.02	97.75	106.52
Secondary CVD	8.19	8.45	8.58	8.37	9.75
Death from other causes	119.95	120.09	119.90	119.58	119.57
Net monetary benefit per patient, mean					
£20,000 WTP threshold	£138,286,583	£138,278,979	£138,273,695	£138,338,768	£138,260,266
£30,000 WTP threshold	£207,717,772	£207,705,699	£207,698,933	£207,797,915	£207,662,457

QALYs are the quality adjusted life years, WTP is the willingness to pay, and discounted costs and QALYs reflect time-preference for current benefits over future ones.

e) Deterministic analyses with 5,000 iterations when cardiovascular event costs are doubled.

Outcomes	Algorithm				
	**General lipid algorithm**	**SMI-specific lipid algorithm**	**General BMI algorithm**	**SMI-specific BMI algorithm**	**No algorithm**
Costs, QALYs and number of events, mean					
Total costs discounted	£1,058,395	£1,059,144	£1,068,608	£1,059,167	£1,089,654
QALY discounted	6,944	6,944	6,944	6,944	6,939
Cost compared to no algorithm	£31,259	£30,510	£21,046	£30,487	
QALY compared to no algorithm	5	5	5	5	
Primary CVD	98.39	98.88	100.04	97.94	106.56
Secondary CVD	8.32	8.41	8.62	8.27	9.78
Death from other causes	119.91	119.94	119.80	120.07	119.72
Net monetary benefit per patient, mean					
£20,000 WTP threshold	£137,828,691	£137,817,831	£137,807,069	£137,820,564	£137,683,766
£30,000 WTP threshold	£207,272,235	£207,256,318	£207,244,908	£207,260,429	£207,070,477

QALYs are the quality adjusted life years, WTP is the willingness to pay, and discounted costs and QALYs reflect time-preference for current benefits over future ones.

f) Deterministic analysis with 5,000 iterations when the utility for severe mental illness is altered to the utility associated with relapse of 0.479.

	**Algorithm**				
**Outcomes**	**General lipid algorithm**	**SMI-specific lipid algorithm**	**General BMI algorithm**	**SMI-specific BMI algorithm**	**No algorithm**
Costs, QALYs and number of events, mean					
Total costs discounted	£555,344	£555,354	£557,254	£558,074	£546,564
QALY discounted	3,833	3,832	3,832	3,833	3,828
Cost compared to no algorithm	£8,780	£8,790	£10,690	£11,510	
QALY compared to no algorithm	5	4	4	5	
Primary CVD	98.57	99.07	100.00	97.64	106.82
Secondary CVD	8.28	8.48	8.45	8.19	9.88
Death from other causes	119.67	119.91	119.87	119.80	119.90
Net monetary benefit per patient, mean					
£20,000 WTP threshold	£76,100,361	£76,088,059	£76,081,687	£76,097,587	£76,012,360
£30,000 WTP threshold	£114,428,213	£114,409,765	£114,401,157	£114,425,417	£114,291,822

QALYs are the quality adjusted life years, WTP is the willingness to pay, and discounted costs and QALYs reflect time-preference for current benefits over future ones.

g) Deterministic analysis with 5,000 iterations when the utility for severe mental illness is altered to the utility of severe mental illness with extra pyramidal symptoms of 0.604.

	Algorithm				
**Outcomes**	**General lipid algorithm**	**SMI-specific lipid algorithm**	**General BMI algorithm**	**SMI-specific BMI algorithm**	**No algorithm**
Costs, QALYs and number of events, mean					
Total costs discounted	£556,527	£555,894	£557,176	£559,607	£545,562
QALY discounted	4,840	4,840	4,839	4,840	4,836
Cost compared to no algorithm	£10,965	£10,332	£11,614	£14,045	
QALY compared to no algorithm	4	4	4	4	
Primary CVD	98.68	99.28	100.01	97.74	106.73
Secondary CVD	8.41	8.44	8.59	8.31	9.78
Death from other causes	119.92	119.82	119.94	120.04	119.78
Net monetary benefit per patient, mean					
£20,000 WTP threshold	£96,238,127	£96,236,757	£96,222,616	£96,235,392	£96,168,074
£30,000 WTP threshold	£144,635,453	£144,633,082	£144,612,512	£144,632,891	£144,524,892

QALYs are the quality adjusted life years, WTP is the willingness to pay, and discounted costs and QALYs reflect time-preference for current benefits over future ones.

h) Deterministic analysis with 5,000 iterations when the effect of statin therapy is reduced to the upper odds ratio value of the 95% confidence interval of 0.8 and 0.89 for coronary heart disease and stroke respectively.

	Algorithm				
**Outcomes**	**General lipid algorithm**	**SMI-specific lipid algorithm**	**General BMI algorithm**	**SMI-specific BMI algorithm**	**No algorithm**
Costs, QALYs and number of events, mean					
Total costs discounted	£573,260	£569,690	£569,217	£575,899	£543,790
QALY discounted	6,942	6,942	6,943	6,944	6,940
Cost compared to no algorithm	£29,470	£25,900	£25,427	£32,109	
QALY compared to no algorithm	2	2	3	4	
Primary CVD	101.64	101.84	102.22	101.09	106.56
Secondary CVD	8.48	8.72	8.82	8.43	9.78
Death from other causes	120.06	120.00	119.78	120.04	119.57
Net monetary benefit per patient, mean					
£20,000 WTP threshold	£138,269,835	£138,275,280	£138,290,040	£138,294,240	£138,246,564
£30,000 WTP threshold	£207,691,382	£207,697,765	£207,719,669	£207,729,309	£207,641,741

QALYs are the quality adjusted life years, WTP is the willingness to pay, and discounted costs and QALYs reflect time-preference for current benefits over future ones.

i) Deterministic analysis with 5,000 iterations when compliance with statin therapy is reduced to 50%.

Outcomes	Algorithm				
	**General lipid algorithm**	**SMI-specific lipid algorithm**	**General BMI algorithm**	**SMI-specific BMI algorithm**	**No algorithm**
Costs, QALYs and number of events, mean					
Total costs discounted	£550,166	£551,821	£552,657	£551,173	£545,588
QALY discounted	6,944	6,943	6,942	6,944	6,939
Cost compared to no algorithm	£4,578	£6,233	£7,069	£5,585	
QALY compared to no algorithm	5	4	3	5	
Primary CVD	100.07	100.80	101.46	99.58	106.63
Secondary CVD	8.37	8.43	8.59	8.27	9.81
Death from other causes	119.90	120.03	119.98	120.16	119.97
Net monetary benefit per patient, mean					
£20,000 WTP threshold	£138,329,253	£138,312,446	£138,290,292	£138,317,735	£138,223,896
£30,000 WTP threshold	£207,768,962	£207,744,580	£207,711,676	£207,752,189	£207,608,638

QALYs are the quality adjusted life years, WTP is the willingness to pay, and discounted costs and QALYs reflect time-preference for current benefits over future ones.
